# Beyond camera presence: individual differences and emotional climate shape conversational memory in online meetings

**DOI:** 10.3389/fpsyg.2026.1794843

**Published:** 2026-10-02

**Authors:** Ya-Jie Tai, Jen-Tang Cheng, Tsung-Ren Huang

**Affiliations:** Department of Psychology, National Taiwan University, Taipei City, Taiwan

**Keywords:** emotional climate, memory, online meetings, personality, social presence

## Abstract

Online meetings have become ubiquitous, yet whether keeping the camera on improves or impairs memory for what is said remains contested. The cognitive-load (Zoom-fatigue) framework predicts impairment, whereas the social-presence framework predicts facilitation. We adjudicated these competing predictions in a within-subjects experiment in which 86 university students conversed dyadically with confederates on a custom WebRTC platform across four conditions: camera-off, and camera-on with positive, neutral, or negative emotional climates. After the conversation, a surprise free-recall test measured memory for the partner’s spoken content. Linear mixed-effects modeling (marginal *R*^2^ = 0.15; conditional *R*^2^ = 0.34) revealed that recall was reliably predicted by sex (*β* = −0.21, *p* = 0.006) and agreeableness (*β* = −0.20, *p* = 0.006), but not by camera presence. Extraversion further interacted with the neutral climate (*β* = −0.13, *p* = 0.021), such that extraverts recalled less when their partner displayed no interpretable socioemotional cues. These findings argue for foregrounding individual differences and emotional context, rather than camera state alone, in models of memory in videoconferenced communication.

## Introduction

Online meetings have become embedded in everyday work, education, and clinical practice (Pokhrel and Chhetri, 2021; Goodell, 2020; Seuling et al., 2023; Dedeilia et al., 2020). A defining and seemingly trivial feature of these meetings is the user’s choice to keep their camera on or off, a decision that varies widely across individuals and contexts. Although social norms encourage participants to keep their cameras on (Zabel et al., 2022), individuals frequently turn them off due to bandwidth constraints (Castelli and Sarvary, 2021), privacy concerns (Bedenlier et al., 2021), or accumulated fatigue (Shockley et al., 2021). However, whether this small ergonomic choice has measurable consequences for what participants remember from a meeting is the question we address here.

Two literatures make opposing predictions about how camera presence affects memory for spoken content. The Zoom Fatigue framework (Bailenson, 2021; Fauville et al., 2023; Shoshan and Wehrt, 2022) holds that constant exposure to a partner’s image—and to one’s own thumbnail—generates cognitive overhead through self-monitoring, gaze management, and continuous nonverbal interpretation. This overhead competes with the linguistic processing required to encode meeting content (Chun and Turk-Browne, 2007) and therefore predicts that camera-on conditions impair memory. The Social Presence framework (Wang et al., 2019; Lanthier et al., 2022), in contrast, argues that visible partners increase arousal, social engagement, and perceived co-presence, all of which support attentional engagement and deeper encoding of conversational content; this account predicts that camera-on conditions facilitate memory. The two predictions are mutually exclusive, yet to our knowledge, no within-subjects study using naturalistic dyadic conversation has directly adjudicated between them.

These competing predictions are unlikely to apply uniformly across people. Two classes of moderators are central. First, emotional climate—the affective tone established by a partner’s nonverbal cues—modulates how socially salient a video stream feels (Holler and Levinson, 2019; Mesman et al., 2009; Vogel et al., 2017). A neutral, expressionless partner (a “poker face”) may be especially distracting because it provides no interpretable social feedback, redirecting cognitive resources toward inferring the partner’s stance. Second, personality traits—particularly extraversion, agreeableness, and dispositions related to social-evaluative concern—shape how strongly individuals attend to and seek social feedback (Lieberman, 2000; Kashdan et al., 2014; Zeichner, 2019; El Haj et al., 2021). Extraverts, who are more attuned to social cues, may be especially susceptible to climate-induced distraction (Boal et al., 2018); agreeable individuals, who tend to accept incoming information without active counterarguing, may encode less of what they hear (Eagly et al., 2000). These trait-level differences imply that camera presence and emotional climate may interact with who is in the meeting rather than producing uniform main effects.

The present within-subjects experiment addresses three questions about memory for conversational content in dyadic online meetings, each tied to a directional hypothesis.

*H1* (Camera presence). We test the two competing accounts above. H1a (Zoom Fatigue): memory for the partner’s spoken content will be lower when the partner’s camera is on than when it is off. H1b (Social Presence): Conversely, camera-on conditions were expected to be associated with better recall performance.

*H2* (Emotional climate). Drawing on the emotion–memory literature (Kensinger and Corkin, 2003) and on evidence that ambiguous affect is cognitively costly (Mesman et al., 2009), we hypothesize that recall will be higher in valenced (positive or negative) climates than in a neutral climate.

*H3* (Personality × Climate). We hypothesize that extraversion will moderate the climate effect: extraverts, who are more disposed to seek and process social feedback (Zeichner, 2019), will show a larger memory cost under the neutral climate than introverts, because the absence of interpretable social feedback diverts more of their cognitive resources.

The contribution of this study is threefold. First, by simulating dyadic online meetings on a custom WebRTC platform that preserves typical interface affordances, we test camera and climate effects under conditions that approximate everyday videoconferencing while retaining experimental control. Second, by employing a within-subjects design crossed with measured personality, we can separate the causal effects of the manipulations from individual-difference moderators that prior between-subjects work has confounded. Third, by directly contrasting Zoom Fatigue and Social Presence predictions, we offer empirical adjudication between two frameworks that have so far developed in parallel without a head-to-head comparison.

## Materials and methods

### Materials

#### Memory materials

For each experimental block, participants were presented with a set of arguments on a predetermined topic, delivered verbally by a confederate. The four topics included: impolite behavior in online meetings, living with COVID-19, legal sanctions for mentally ill criminals, and preferential treatment for aboriginal students. For each topic, the study authors pre-selected 10 arguments—five supportive and five opposing—to serve as memory items. These arguments were presented as an on-screen script that the confederate read verbatim. The four scripts were comparable in memorability, with similar lengths (averaging 955.75 ± 77.91 Chinese characters) and a similar number of items recalled by participants in a pilot study.

#### Questionnaires

Narcissists may be overly preoccupied with their appearance (Raskin and Terry, 1988) and focus primarily on themselves during online meetings. We measured participants’ levels of narcissism by the 16-item Short Measure of Narcissistic Personality Inventory (Ames et al., 2006) and the 7-item narcissism subscale from the Short Dark Tetrad (Paulhus et al., 2020). All questionnaires were administered in Mandarin Chinese.

People with low self-esteem may be overly sensitive to a hostile emotional climate (Wang et al., 2021) and pay more attention to others’ body language than to their speech content. In this study, we measured participants’ self-esteem using the 10-item Rosenberg Self-Esteem Scale (Rosenberg, 1979).

Personality traits, particularly extraversion and neuroticism, are critical predictors of behavior and responses in social settings (Shen et al., 2015), such as online meetings (Zabel et al., 2022). We measured participants’ Big Five personality traits (Openness to new experiences, Conscientiousness, Extraversion, Agreeableness, and Emotional Stability) using the Ten-Item Personality Inventory (Gosling et al., 2003).

We also employed other measures of sociability in addition to extraversion. They were the 20-item Social Phobia Scale and the 20-item Social Interaction Anxiety Scale (Mattick and Clarke, 1998). The former measures a participant’s fear of being closely examined during ordinary activities, and the latter measures a participant’s fear of general social interactions, aligning with the DSM classifications of Social Phobia.

Finally, friendship is known to modulate social interaction (Planalp and Benson, 1992) and is thus controlled for in this study. We used the Inclusion of Other in the Self Scale (Aron et al., 1992) to assess friendships, as reported by both a confederate and a paired participant.

#### Interaction environment

The interaction environment is a web platform designed for this experiment that closely mirrors typical online meeting environments while facilitating experimental manipulations. Specifically, the platform was constructed using standard web technologies, including HTML, JavaScript, and CSS. Video-based communication was implemented via Web Real-Time Communication (WebRTC).

However, unlike in other online meeting environments, participants’ screen views differ from those of confederates. In the experimental condition in which a confederate followed an on-screen instruction to turn *off* the camera, a participant saw only their own image on the left side of the computer screen; the paired confederate saw either a script to be read aloud or an instruction to provide nonverbal feedback while listening. In the experimental condition where a confederate’s camera was *on*, the screens viewed by the confederate and the participant were identical to those described above, except that the confederate was visible on the right-hand side of a participant’s screen and at the bottom of the confederate’s screen.

### Actual participants

To detect a correlation greater than 0.3 between two continuous variables at an α level of 0.05 and a power of 0.8, at least 84 samples are needed, as indicated by a power analysis in G*Power 3.1 (Faul et al., 2009). A total of 88 university students were recruited for the study, but two of them who did not follow our experimental procedure were excluded. In the end, 86 individuals (44 females; mean age = 20.52 ± 1.37) were included in the final sample.

To recruit a relatively homogeneous group of dyadic samples, we employed the following recruitment criteria. Participants needed to be enrolled in college at the time, be native Mandarin Chinese speakers, have no reading or speech impairments, have had an acquaintance for 1 to 3 years (excluding intimate partners or extended family), and have been in contact at least once in the past 6 months. These criteria also help to ensure a smooth conversation between a participant and a confederate.

### Confederate participants

Confederate partners were used to standardize spoken content across participants while permitting systematic manipulation of nonverbal emotional cues—a design approach common in social-cognition research (e.g., Mesman et al., 2009). Because one may not mind a total stranger’s opinions or feelings during a conversation, we solicited student confederates who then recruited their friends to participate. The solicitation, including payment information (~$87 USD for 16 working hours), was announced in a Facebook group widely used by university students. In the end, 14 undergraduate students from six colleges (7 females; age = 20.79 ± 1.19) volunteered to serve as confederates. These confederates then underwent a pre-study training session to learn the experimental procedure.

The confederates were instructed to converse with a participant in a turn-taking manner throughout the experiment and to create different emotional climates using perceivable body language across blocks. In each block with the camera on, when it was a participant’s turn to speak, the confederate followed on-screen instructions to provide a randomly chosen type of emotional feedback (negative, neutral, or positive). The operational definitions of these three emotional climates were as follows: a positive climate involved nodding, smiling, and a slight forward lean; a neutral climate involved a still face with no nodding (i.e., a “poker face”); and a negative climate involved head shaking, frowning, and a slight backward lean. When it was a confederate’s turn to speak, the confederate read from an on-screen script in a natural way.

After being introduced to the procedure, each confederate completed approximately 3.5 h of structured training, including a standardized demonstration and supervised practice with an experimenter using a shortened version of the experiment. Upon completing the training hours, each confederate underwent a dual-examiner certification before being approved for formal data collection: one examiner served as the video-call partner to evaluate whether the confederate’s facial expressions and eye-gaze patterns were naturalistic and not transparently scripted, while a second examiner sat alongside the confederate to assess vocal tone consistency and script-reading fluency. Confederates were required to satisfy both examiners before proceeding to data collection. Confederates were aware of the study’s general framing as a behavioral experiment on online communication, but were not informed of the specific hypotheses regarding memory performance.

After the pre-study training, each confederate began inviting individuals from their social networks who met the recruitment criteria to serve as research participants, aiming for 6 to 8 accepted invites. During the invitation, the study’s purpose was kept confidential from the participants. After the invitation, a confederate joined the formal experiment with each participant at a time convenient to both parties.

Before formal data collection, two pilot sessions were conducted with N = 12 independent participants, whose data were not included in the final analyses. The pilot study served three purposes: (a) to verify the technical stability of the custom WebRTC video platform, (b) to evaluate the suitability and comparability of the memory scripts across the four topics, and (c) to train the experimenters in protocol administration. The scripts used in the formal study were not modified as a result of the pilot.

### Experimental procedure

The design and procedure of this study were approved by the Research Ethics Committee of National Taiwan University. The study took approximately 2 h to complete, and each participant (including the confederate) was paid approximately 11 USD for their participation. After the experimenters described the study procedure to the participants, all participants provided written informed consent before participating. Specifically, participants were informed that the study would examine behavioral patterns in online meetings and that video recording would enable *post hoc* analysis of the interaction. In other words, the participants were unaware of the research’s true purpose and of the surprise memory test at the end of the study.

One pair of participants—one actual and one confederate—was invited to each experimental session. The study began with two experimenters explaining the details of the experiment to both the confederate participant and the actual participant. Once the introduction was completed, each participant was led by an experimenter to a separate room, where they sat in front of a laptop with a camera. Both participants read the experiment instructions on their computer screens before proceeding to a chat block.

The experimental structure is illustrated in Figure 1. There were four chat conditions in total, with four predetermined topics randomly chosen by the experimenter before each condition. Each chat condition consisted of two turns. The first turn was always an actual participant sharing thoughts on a given topic so that the confederate could create an emotional climate. Specifically, as the participant spoke, the confederate, following the on-screen instructions, would change the video status and the type of nonverbal feedback. After the participant finished sharing, it became the confederate’s turn to speak (i.e., read the on-screen script aloud).

**Figure 1:**
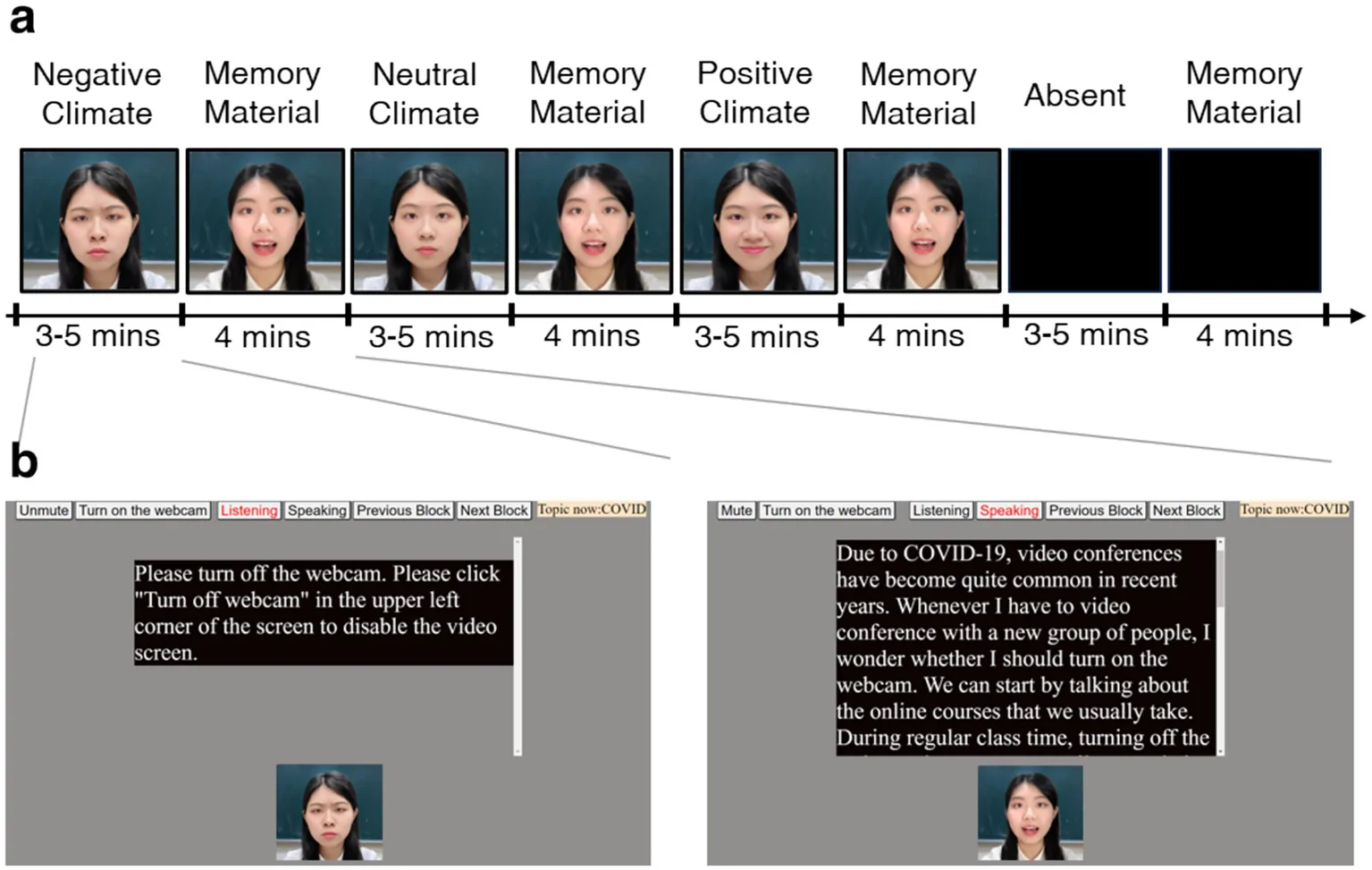
Research overview. This is an overview of the experimental design, divided into three distinct panels. **(a)** The upper panel illustrates the perspective of an actual participant seeing the confederate posing in four different experimental conditions. **(b)** The lower panel illustrates the perspective of a confederate seeing on-screen instructions (left) or scripts (right). Figure contains images of the author(s) only.

By design, the four implemented conditions were Camera-Off, Camera-On + Positive, Camera-On + Neutral, and Camera-On + Negative. Because emotional climate was conveyed exclusively through visible nonverbal cues, the design entailed a structural asymmetry: camera presence is fully crossed with the availability of socioemotional cues, and the present analyses cannot estimate the effect of camera presence independently of climate-cue availability. A fully crossed 2 × 3 design—for example, with prosodic conveyance of emotional climate in the camera-off condition—would be required to isolate the independent effect of camera presence.

After all chat conditions concluded, participants completed a series of questionnaires and underwent a surprise memory test. As described in the *Materials* section, the questionnaires assessed personal characteristics related to socioemotional processing. They took about 10 min in total. The surprise memory test measured how much of the conversation content each participant remembered. It took approximately 10 min for each participant to freely recall, as much detail as possible, what the other (confederate) participant had said. The participant’s verbal responses in each of the four chat conditions were recorded and later scored by the experimenters, yielding four free-recall scores per participant.

### Statistical analysis

Because the four free-recall scores for each participant were likely correlated, we used mixed-effects models rather than multiple regressions to investigate which factors accounted for participants’ memory performance. Each mixed-effects model included a fixed slope and a random intercept. The fixed slopes were estimated across participants to capture linear relationships between a response variable (i.e., recall rate) and all explanatory variables, such as experimental conditions (i.e., camera off or on, with different emotional climates) and personal characteristics (e.g., age, sex, personality). Random intercepts were estimated for each participant to account for individual differences in memory capacity.

This study used various R packages for data cleaning, manipulation, and modeling. The mixed-effects models were implemented using the *lme4* package (Bates et al., 2014), with *p*-values calculated with the *lmerTest* package (Kuznetsova et al., 2017) and *R*^2^ calculated with the *sjPlot* package (Lüdecke, 2021).

Two complementary aspects of the model deserve emphasis. First, camera presence and emotional climate were manipulated within subjects, so their estimated coefficients are interpretable as causal effects within the limits of the present design. In contrast, personality, sex, age, and friendship were measured rather than manipulated, so their coefficients are best described as associations rather than causal effects and require independent replication. Second, to avoid the appearance of significance-based variable selection, the primary model reported in the main text was specified *a priori* on theoretical grounds (extraversion, agreeableness, conscientiousness, the three climate dummies, and four control variables).

To support transparency and robustness, the Supplementary materials include three additional sets of analyses. Section S1 reports three sensitivity analyses addressing the recency effect. S2 presents model diagnostics, including residual normality and variance inflation factors. S3 provides a coefficient plot summarizing the standardized fixed-effect estimates.

## Results

We first conducted a manipulation check of the experimental conditions, followed by a quality check of participants’ free recall scores. To confirm whether each participant paid visual attention to the other participant (the confederate) when the camera was on, the two experimenters reviewed each recorded experimental session and coded each participant’s gaze target into three categories (self, partner, or other) every 10 milliseconds. The inter-rater agreement of the gaze-coding in terms of Krippendorff’s α was 0.941. The coding results show successful manipulation, as participants frequently gazed at their paired partners when the camera was on (see Figure 2). This finding also confirms that participants were visually attending to the confederate’s nonverbal displays — a necessary precondition for the emotional-climate signals to be received. As for the scoring of participants’ free recalls, the inter-rater agreement, measured by Krippendorff’s α, was 0.794. The coded recall rate for each block/condition was then used as the response variable in two mixed-effects models.

**Figure 2:**
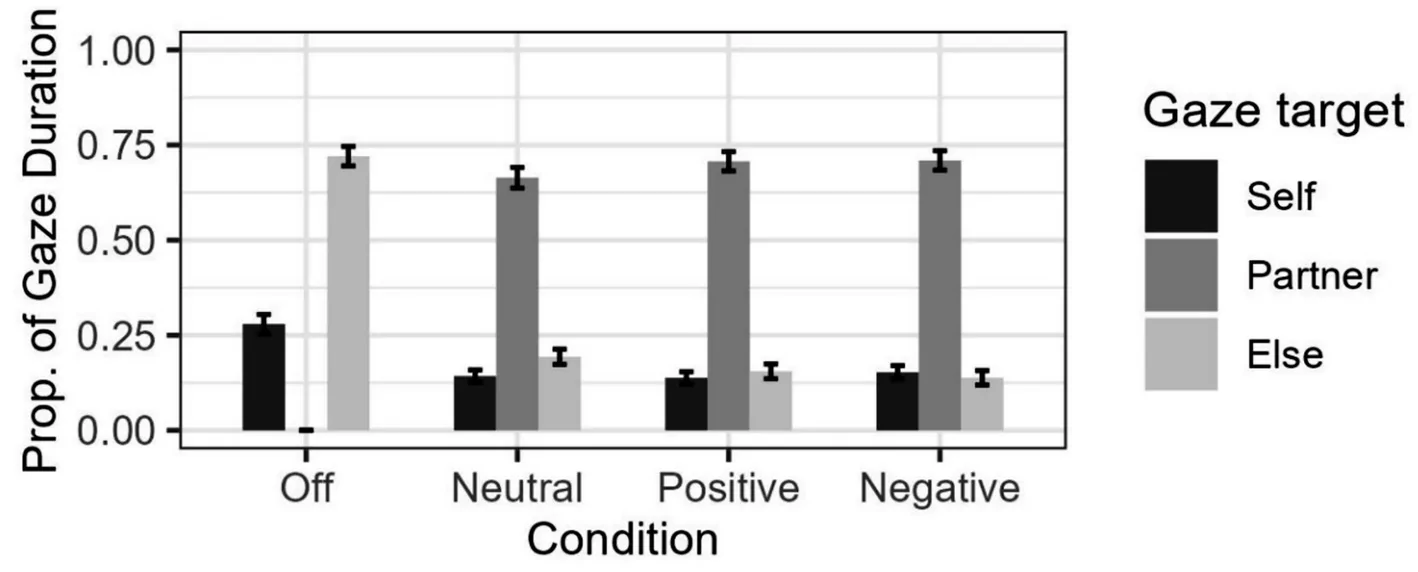
Proportion of gaze duration in four different experimental conditions. The results confirm the success of our manipulation—participants indeed spent more time gazing at their conversation partners than themselves when the partners turned on cameras.

Preliminary analyses showed no significant associations between the measured individual-difference variables and free recall performance. Specifically, social anxiety (assessed via the Social Interaction Anxiety Scale [SIAS] and the Social Phobia Scale [SPS]), narcissism (assessed via the Short Dark Tetrad [SDT]) and the Narcissistic Personality Inventory [NPI], and self-esteem (assessed via the Rosenberg Self-Esteem Scale [RSES]) were examined as potential predictors in separate multilevel models controlling for sex, age, relationship closeness, and condition order. None of these variables significantly predicted free recall performance (SIAS: *β* = −0.005, *p* = 0.944; SPS: *β* = −0.014, *p* = 0.846; RSES: *β* = 0.060, *p* = 0.404; SDT: *β* = 0.080, *p* = 0.254; NPI: *β* = 0.062, *p* = 0.371).

The results of the two mixed-effects models are summarized in Table 1. Both linear mixed models include fixed-effect slopes and random-effect intercepts. The fixed slopes are similar in meaning to the slopes in a multiple regression, where standardized coefficients are effect sizes. The random intercepts partition the variance unexplained by the fixed slopes into the between-individual variance of intercepts (τ_00_) and the residual within-individual variance (σ^2^). The intra-class correlation (ICC) quantifies the amount of variation in the dependent variable attributed to between-individual variance (τ_00_/[σ^2^ + τ_00_]), with values between zero and one. When ICC is 0, individual sample grouping is not required, and the data can be analyzed using multiple regression. Marginal *R*^2^ measures the percentage of total variance explained by the fixed effects, whereas conditional *R*^2^ measures the percentage of total variance explained by both the fixed and random effects.

**Table 1:** Two mixed-effects models of free recall rates.

Predictor	Base model			Interaction model		
	*β*	95% CI	*p*	*β*	95% CI	*p*
Fixed effects						
Condition order	0.22	0.12–0.31	**<0.001**	0.20	0.11–0.29	**<0.001**
Sex ^a^	−0.21	−0.35 to −0.06	**0.006**	−0.21	−0.35 to −0.06	**0.006**
Agreeableness (A)	−0.20	−0.33 to −0.06	**0.006**	−0.20	−0.33 to −0.06	**0.006**
Extraversion (E)	0.10	−0.03 to 0.23	0.140	0.10	−0.03 to 0.23	0.140
Age	−0.08	−0.21 to 0.06	0.265	−0.08	−0.21 to 0.06	0.265
Friendship	−0.06	−0.20 to 0.07	0.376	−0.06	−0.20 to 0.07	0.376
Neutral Climate (Neu)	−0.06	−0.17 to 0.05	0.294	−0.06	−0.17 to 0.05	0.312
Positive Climate (Pos)	0.04	−0.07 to 0.15	0.457	0.04	−0.07 to 0.15	0.453
Conscientiousness (C)	−0.04	−0.17 to 0.10	0.608	−0.04	−0.17 to 0.10	0.608
Negative Climate (Neg)	−0.03	−0.14 to 0.08	0.577	−0.03	−0.14 to 0.08	0.570
E × Neu				−0.13	−0.24 to −0.02	**0.021**
C × Neu				−0.07	−0.18 to 0.04	0.222
A × Pos				−0.05	−0.16 to 0.06	0.377
A × Neg				0.05	−0.06 to 0.16	0.404
C × Pos				−0.04	−0.15 to 0.07	0.445
C × Neg				−0.02	−0.13 to 0.09	0.731
A × Neu				0.02	−0.09 to 0.13	0.711
E × Neg				0.01	−0.10 to 0.12	0.849
E × Pos				−0.01	−0.12 to 0.10	0.879
Random effects						
σ^2^	0.02			0.02		
τ_00_	0.01			0.01		
ICC	0.22			0.22		
N participants	86			86		
Observations	344			344		
Marginal R^2^	0.126			0.149		
Conditional R^2^	0.314			0.336		

Predictors are sorted by their effect sizes regardless of significance. Those with *p*-values smaller than 0.05 are highlighted in bold. ^a^Sex was coded 1 = male, 0 = female; σ^2^ = residual variance; τ_00_ = variance of intercepts; ICC = intraclass correlation coefficient.

The first model is a base model without interaction terms. There are three groups of explanatory variables. The first group consists of the experimental conditions (i.e., camera on or off across different emotional climates). The second group consists of personality predictors specified *a priori* on theoretical grounds: extraversion, agreeableness, and conscientiousness. Extraversion and agreeableness were selected because prior theory and the study’s hypotheses directly implicate them in socioemotional processing during conversation; conscientiousness was included as a theoretically adjacent trait that influences self-regulation and attentional control. The third group consists of control variables: age, sex, friendship (IOS), and condition order. Condition order is a binary variable coded as 0 for the first half of the blocks and 1 for the second half, to account for the well-known recency effect in memory.

As shown in Table 1, the base model indicates that the experimental conditions do not account for participants’ memory performance, whereas some control variables and personal characteristics do. Specifically, among the four control variables, only condition order and sex showed statistically significant relationships with memory performance. Participants recalled more items in later blocks than in earlier ones, and male participants recalled fewer items than female participants. These results are consistent with the recency effect and sex effect in the memory literature (Lanthier et al., 2019). As for the three personal characteristics, only agreeableness was significantly associated with memory performance. More agreeable individuals recalled less of the content they heard.

As shown in Table 1, the full model also includes interactions between the experimental conditions and personal characteristics. This model is designed to investigate whether the null effect observed in the first model was due to the mixing of opposing effects from individuals with different personal characteristics. The overall results of the full model are similar to those of the base model. Among all the explanatory variables, condition order, sex, and agreeableness are the most predictive of memory performance. Additionally, the interaction between extraversion and the neutral climate condition has a statistically significant negative relationship with memory performance. In other words, when extroverts were in a neutral climate, they tended to remember less than in conditions where the camera was off or on, with positive or negative climates.

Model assumptions were checked using diagnostic plots. The base and full models showed no detectable violations of normality (Shapiro–Wilk: Base W = 0.997, *p* = 0.76; Full W = 0.996, *p* = 0.44), and no excess outliers. Variance inflation factors were below 1.6 for the base model. In contrast, in the full interaction model, the inflated VIF values for the climate dummies and their personality interactions reflect the structural correlation between dummy main effects and their interactions in an uncentered factorial specification (mean-centering the personality predictors would mechanically reduce these VIFs without altering the inferences).

## Discussion

In a within-subjects experiment with four implemented conditions (camera-off and camera-on with positive, neutral, or negative emotional climates), recall of conversational content was reliably predicted by sex, agreeableness, and the interaction between extraversion and a neutral climate, but not by camera presence per se. Participants higher in agreeableness recalled less of the conversation content; one possible mechanism is that more agreeable individuals counterargued less and thus encoded the message less deeply (Eagly et al., 2000; Huang and Hsieh, 2024), although the present study did not directly test this. Extraverts recalled less content under the neutral climate (*β* = −0.13, *p* = 0.021), consistent with prior reports that extraverts are more sensitive to social feedback during memory and cognitive tasks (El Haj et al., 2021; Lieberman, 2000).

### Interpreting the camera contrast

The camera contrast yielded no reliable association with recall performance. Three considerations bear on how this null should be interpreted. First, emotional climate in the present design was conveyed exclusively through visible nonverbal cues, so camera presence and socioemotional cue availability were varied together rather than independently; the present analyses cannot isolate the effect of camera presence from that of cue availability, and a fully crossed 2 × 3 design—with prosodic conveyance of climate in the camera-off condition—would be required to do so. Second, the power analysis was optimized to detect medium-sized correlations, and the present design has limited sensitivity to small camera effects. Third, the two theoretical accounts most frequently cited in the literature—Zoom Fatigue (Bailenson, 2021; Fauville et al., 2023; Shoshan and Wehrt, 2022) and Social Presence (Wang et al., 2019; Lanthier et al., 2022)—generate opposing directional predictions about camera effects on memory; the present null is therefore consistent with either account being only partially operative under the specific conditions of brief, structured dyadic conversations, and does not adjudicate between them. None of these considerations rules out a camera effect; they collectively caution against treating the observed null as evidence that camera state is irrelevant to memory in general.

### Personality and emotional climate

Recall was lower under the neutral climate, particularly for extraverts. Infants and adults alike show heightened arousal when a partner displays a still, expressionless face (Mesman et al., 2009), because the absence of interpretable nonverbal feedback complicates inferences about the partner’s stance. Under such conditions, participants may divert cognitive resources toward inferring the social situation, leaving fewer resources available for encoding spoken content. Extraverts, who tend to seek and process social feedback more actively (Zeichner, 2019), may be especially susceptible to this redirection. This account is consistent with the observed Extraversion × Neutral interaction; the interaction itself is robust to excluding the final block of trials and to substituting a continuous block-order covariate for the binary recency term (Supplementary materials, Section S1), but loses statistical significance when the analysis is restricted to the first two blocks because the effective sample is halved (*p* = 0.093), likely reflecting reduced power rather than a genuine moderation of the effect.

### Theoretical implications

Two implications follow. First, individual differences and emotional context, rather than camera state alone, may be the more productive level of analysis for memory in videoconferenced communication (El Haj et al., 2021; Kensinger and Corkin, 2003; Lieberman, 2000). Second, neither the Zoom Fatigue (Bailenson, 2021; Fauville et al., 2023; Shoshan and Wehrt, 2022) nor the Social Presence framework (Lanthier et al., 2022; Wang et al., 2019), as applied here, fully accounts for the pattern of results: the absence of a reliable camera contrast is consistent with each framework being only partially correct, with no decisive empirical adjudication available under the present design. We view our findings as constraining future designs more than as resolving the theoretical conflict; in particular, future tests should decouple camera presence from the availability of socioemotional cues and should incorporate direct measures of cognitive load and arousal.

### Future directions and tentative implications

The present findings are best understood as a first step toward a broader research agenda. The within-subjects design using brief, structured dyadic conversations among Taiwanese university students offers strong internal validity but naturally constrains generalizability. Future work should extend these questions to workplace professionals, older adults, and cross-cultural settings, where the cognitive and social demands of camera presence may operate differently (Fauville et al., 2023; Shoshan and Wehrt, 2022), and to longer, multi-party meetings that more closely approximate everyday videoconferencing.

Future studies would benefit from post-trial manipulation-check ratings or independent blind coding of recorded sessions to quantify perceived emotional climate directly. More granular free-recall rubrics or semi-automated scoring would strengthen measurement precision. Retaining confederate identifiers as a permanent variable would allow confederate-level random effects to be modeled, addressing a source of non-independence not captured in the present analyses.

The marginal *R^2^* values of 0.13 (Base model) and 0.15 (Full model) indicate that substantial variance remains unexplained, pointing to unmeasured constructs—working memory capacity, cognitive load, and fatigue—as productive targets for future work (RepovŠ and Baddeley, 2006; Moran, 2016; Unsworth and Miller, 2021; Robison and Brewer, 2022). The conditional *R^2^* values of 0.31 and 0.34 confirm that stable individual differences account for a meaningful share of variance, reinforcing the study’s individual-differences focus. Incorporating validated measures such as the Operation Span Task or NASA-TLX would allow the unique contributions of camera presence and emotional climate to be isolated above and beyond general cognitive resources. Whether training agreeable individuals in active processing, eliminating poker-faced presentations, or accounting for introvert–extravert differences in cognitive cost improves memory outcomes are questions that intervention studies are better positioned to answer.

## Data Availability

The datasets presented in this study can be found in online repositories. The names of the repository/repositories and accession number(s) can be found at: https://osf.io/qkc24.
