# Supplementary material for: Beyond camera presence: individual differences and emotional climate shape conversational memory in online meetings
Source: Front Psychol. 2026 Oct 2;17:1794843. doi: 10.3389/fpsyg.2026.1794843 (PMC13672515; doi:10.3389/fpsyg.2026.1794843)
Supplement: Supplementary file 1 [file Supplementary_file_1.docx]

**Beyond Camera Presence: Individual Differences and Emotional Climate Shape Conversational Memory in Online Meetings**

**Section S1: Sensitivity Analyses**

The main effects of sex and agreeableness, and the Extraversion × Neutral interaction, might be driven by the recency effect (i.e., higher recall in the final block of trials due to its proximity to the surprise free-recall test). To address this concern, we conducted three complementary sensitivity analyses. In Sensitivity A, we refit the principal mixed-effects model after excluding observations from each participant's final block (one observation per participant; 86 observations removed; n = 258). In Sensitivity B, we restricted the analysis to each participant's first two blocks (n = 172). In Sensitivity C, we replaced the binary recency covariate (first half = 0; second half = 1) with a continuous, z-scored block-order index ranging from 1 to 4. All three sensitivity analyses used the same predictors as the theory-driven principal model (Extraversion × Climate interactions, Agreeableness, Conscientiousness, sex, age, IOS, and recency) and were estimated by restricted maximum likelihood (REML) with *lme4::lmer* and Satterthwaite-approximated degrees of freedom (*lmerTest*).

Table S1 reports the three flagship effects (sex, agreeableness, and the Extraversion × Neutral interaction) across the principal model and the three sensitivity analyses, as well as the overall model fit indices (number of observations and marginal R²). The two main effects (sex and agreeableness) were preserved in both directions and statistically significant across all four specifications. The Extraversion × Neutral interaction was also preserved in direction (standardized β between −0.11 and −0.15) and remained statistically significant in Sensitivity A (*p* = .031) and in Sensitivity C (*p* = .046). When the analysis was restricted to the first two blocks (Sensitivity B), the effective sample size was halved by design, and the interaction was no longer statistically significant (*p* = .093), as expected given the reduced power to detect small effects.

**Table S1.** Three effects across the principal model and three sensitivity analyses.

| **Effect** | **Revised principal model (full data)** | **Sensitivity A:**  **drop block 4** | **Sensitivity B:**  **first half only** | **Sensitivity C:**  **order continuous** |
| --- | --- | --- | --- | --- |
| Sex (std β [95% CI], p) | −0.21 [−0.35, −0.06]  ***p* = .006** | −0.23 [−0.40, −0.07]  ***p* = .006** | −0.26 [−0.45, −0.06]  ***p* = .010** | −0.21 [−0.35, −0.06]  ***p* = .006** |
| Agreeableness  (std β [95% CI], p) | −0.20 [−0.33, −0.06]  ***p* = .006** | −0.23 [−0.38, −0.07]  ***p* = .004** | −0.23 [−0.41, −0.04]  ***p* = .015** | −0.20 [−0.33, −0.06]  ***p* = .005** |
| Extraversion × Neutral  (std β [95% CI], p) | −0.13 [−0.24, −0.02]  ***p* = .022** | −0.15 [−0.29, −0.01]  ***p* = .031** | −0.14 [−0.31, 0.02]  *p* = .093 | −0.11 [−0.21, .00]  ***p* = .046** |
| Observations  Participants | 344  86 | 258  86 | 172  86 | 344  86 |
| Marginal R² | 0.141 | 0.124 | 0.117 | 0.164 |

**Note.** Standardized betas for sex and agreeableness are computed within each fitted dataset using β × SD(x) / SD(y); 95% confidence intervals are Wald-based on the standardized scale. Bold *p*-values indicate *p* < .05. The continuous block-order specification (Sensitivity C) yielded a marginally higher marginal *R*² (.164) than the binary recency specification (.141) in the principal model, supporting the order-related variance captured by the block-order index.

**Section S2: Model Diagnostics**

We assessed the assumptions of the linear mixed-effects models using five complementary diagnostic procedures applied to the Base and Full models. (1) A singular fit was inspected using the *lme4* package. (2) Residual normality was tested by the Shapiro–Wilk test. (3) Variance inflation factors (VIF) were computed for each model. (4) Influential observations were screened via Cook’s-distance diagnostics (threshold 4/n). (5) Visual residual diagnostics (Q–Q of residuals; residuals vs. fitted) were generated using base R graphics. The numerical results are summarized in Table S2; the residual diagnostic plots appear in Figures S1 and S2.

**Table S2.** Numerical diagnostic results for the Base and Full linear mixed-effects models.

| **Diagnostic** | **Base model** | **Full model** |
| --- | --- | --- |
| Singular fit | FALSE | FALSE |
| Shapiro–Wilk W  (residual normality) | 0.997, *p* = .76 | 0.996, *p* = .44 |
| Cook's-distance influential observations | 0 (Cook's 4/n) | 0 (Cook's 4/n) |
| Visual Q–Q of residuals | Q–Q plot near linear | Q–Q plot near linear |
| Visual residual-vs-fitted pattern | no systematic  mean–variance pattern | no systematic  mean–variance pattern |
| VIF - main predictors (max) | 1.54 (neu) | 2.56 (conscientiousness) |
| VIF - interaction terms (max) | — | 46.79 (climate dummies) |

**Note.** All test-based diagnostics yielded non-significant results at α = 0.05, and Cook’s distance screening flagged no influential observations in either model, indicating no detectable violations of the linear mixed-effects assumptions. The inflated VIFs in the Full model arise from the well-known structural correlation between dummy main effects and their interactions in an uncentered factorial specification; mean-centering the personality predictors would mechanically reduce these VIFs without altering inferences. The visual residual diagnostics (Q–Q plots and residuals vs. fitted values) confirmed that the inflated VIFs do not indicate real model misspecification.

Figure S1 shows the residual diagnostic plots for the Base model; Figure S2 shows the same diagnostics for the Full model. Both figures display two panels: (left) a Q–Q plot of model residuals against theoretical quantiles; (right) residuals vs. fitted values. Across both models, the Q–Q lines are approximately linear, and the residual-vs-fitted plots show no systematic pattern, consistent with the Shapiro–Wilk results in Table S2 and confirming the absence of detectable residual misspecification.

**Figure S1.** Residual diagnostic plots — Base model. Left: Q–Q plot of model residuals against theoretical quantiles. Right: residuals vs. fitted values.

**Figure S2.** Residual diagnostic plots — Full model. Left: Q–Q plot of model residuals against theoretical quantiles. Right: residuals vs. fitted values.

**Section S3: Fixed-Effects Coefficient Plot**

Figure S3 displays the standardized β estimates and 95% Wald confidence intervals for all predictors in the Base Model, estimated via restricted maximum likelihood (REML) with Satterthwaite-approximated degrees of freedom. Predictors are organized into three panels: Experimental Condition (positive, neutral, and negative climate relative to camera-off), Personality (Extraversion, Agreeableness, and Conscientiousness), and Control Variables (sex, age, friendship closeness [IOS], and condition order). Coefficients whose confidence intervals do not include zero are shown in red (p < .05); those that do are shown in grey (n.s.). The plot makes it immediately apparent that sex (β = −0.21, *p* = .006) and Agreeableness (β = −0.20, *p* = .006) emerged as significant predictors of free-recall rate. In contrast, the three experimental conditions did not differ significantly from the camera-off baseline.

**Figure S3.** Fixed-effects coefficient plot for the Base Model. Standardized β estimates and 95% Wald confidence intervals are displayed for all predictors, organized into three groups: Experimental Condition, Personality, and Control Variables. Red-filled circles indicate *p* < .05; open grey circles indicate non-significant predictors (n.s.). The model was estimated with REML via *lme4::lmer* with Satterthwaite degrees of freedom (*lmerTest*).
